# An Account of a Certain and Rapid Method of Curing Syphilis by Preparations of Iodine

**Published:** 1845-04

**Authors:** 


### Art. XI.

*Darstellung einer sicheren und schnellen Heilmethode der Syphilis durch Jodpräparate.* Von Dr. Georg Moj'sisovics.—*Wien*, 1845.

*An Account of a certain and rapid method of curing Syphilis by Preparations of Iodine.* By Dr. G. Moj'sisovics.—*Vienna*, 1845. 8vo.

From the title of this work, and from some passages in the author's preface, we should be led to imagine that it was merely a practical account of the treatment of syphilitic affections, pointing out some decidedly new and certain line of practice in the disease in question. It contains, however, also a full description of the various preparations of iodine as well as the writer's views respecting the nature and pathology of syphilis, (under which title he also includes gonorrhea), the treatment of strictures of the urethra, of primary and secondary syphilis, and a few cases to illustrate his remarks. In the present notice we shall confine ourselves principally to the practical part of the treatise.

Dr. Moj'sisovics' field of observation in the treatment of disease by iodine and other means has been sufficiently extensive to give considerable credit to any work of his upon practical subjects. He is one of the principal surgeons in the large general hospital in Vienna, and is also employed in private practice in that city, in which scrofulous diseases, and others requiring the use of iodine, and usually treated by that remedy, are peculiarly prevalent. In his preface he expresses a hope that his work will be welcome to the medical profession from its containing " a description of a plan of treatment by means of which the most obstinate and inveterate cases of syphilis can be radically cured within three or four weeks, by the careful use of the preparations of iodine;" and if this is so, he certainly has a right to hope that his book will be welcome.

Dr. Moj'sisovics had, for many years, been in the habit of employing iodine in various forms and with very variable success, and being dissatisfied with the want of consistency in the effects of his remedy, he endeavoured, but for a long time in vain, to discover the cause of his occasional failure. Having, as he considered, at last discovered it, he gave an account of the result of his investigations in a paper read before the Imperial Society of Physicians in Vienna, in Oct. 1839, which was printed in the 'Austrian Journal' for the month of March 1840. This paper, he has reprinted in the commencement of the present work without any alteration. It is entitled " The determination of the circumstances which render the effects of iodine curative or injurious." The chief causes of the disappointment which Dr. Moj'sisovics so often met with in the effects of what he terms a specific remedy in cases of scrofulosis and syphilis, were impurity of the drug, and its administration in an improper form; and the injurious consequences which occasionally followed when the medicine was in a pure state were, according to his views, owing to decomposition even after it was swallowed. For a certain amount of the disrepute into which it fell, he blames Lugol who allowed his patients to walk about during the treatment, and paid but slight attention to regimen. Our author says that " this may perhaps do for Frenchmen, but when a German has caries in any joint, it swells, becomes inflamed, painful, changed in form, stiff or contracted, and any attempt at motion is exceedingly painful."

The chapter entitled " Pharmacology of the preparations of iodine," contains a description of thirteen varieties: viz. pure iodine, the tincture, amylum iodatum, the iodides of barium, iron, lead, sodium and potassium, the iodide and biniodide of mercury, the ioduretted syrup of iron, iod-ammonium and the various mineral waters of which this body is an ingredient. The author objects to iodine in a pure state, and to the tincture, on account of the insolubility of the substance itself when it is precipitated upon the lining membrane of the mouth, pharynx, or stomach, and to the latter (the tincture) he attributes the greater share of the disrepute into which the remedy has occasionally fallen. In conjunction with the iodide of potassium, iodine is sufficiently soluble to render it a serviceable form in which it may be administered. The amylum iodatum deserves no mention with reference to its practical importance, and the iodide of barium in the only case in which he administered it produced hemorrhage from the uterus, undoubtedly caused by the medicine, for it recurred several times immediately upon its resumption. The iodide of iron was found exceedingly useful in some cases of chlorosis, and leucorrhea, administered internally, and in the form of baths; it is easily decomposed, and on this account there is an inconvenience in its exhibition. The following extract giving an account of a mineral spring containing this salt is curious:

"In Hungary, on the borders of Styria and Austria, is a mineral spring formerly known to the Romans, the water from which has been used for a very long time as a bath and as an internal remedy. The village in which it appears is called Tazmannsdorf, and there is a well-appointed establishment there. For a long while the water was considered to contain merely carbonate of iron, but chemical analysis has proved the existence of a quantity of iodide of sodium and oxide of iron, besides many bicarbonated salts. By this natural union of iodine and iron the powerful effect of the water upon the nutrition, and upon a low or enfeebled condition of the nervous system is easily explained. The beautiful scenery in the neighbourhood and the inexpensive life, render it a still more desirable spot."

The iodides of lead and sodium have been too little tried by the author, to enable him to speak with certainty respecting their remedial use, but the iodide of potassium (kali hydrojodicum, or kalii bijodetum) has been tried by all, and by Dr. Moj'sisovics is considered to be by far the best of all the preparations of iodine. Its effects are said to be increased activity and power in the function of digestion, from its operation on the mucous membrane of the alimentary canal, and afterwards by an extension of a similar action, a state of the Schneiderian membrane comes on, exactly resembling a common catarrh. "This appearance is favorable, as it proves that the individual is susceptible of the powers of the remedy." The dose usually recommended, merely for increasing the power of the absorbent vessels, is a half grain of iodine, with two or three grains of iodate of potassium, in a considerable quantity of water taken before dinner. In syphilis and scrofulosis, larger doses may be given: an infant, for instance, had five grains, and a child, six years old, began with half a scruple, increasing three grains every fourth day, up to half a drachm. At the age of fifteen the dose at which we may commence is a scruple, gradually increased to a drachm, even to two.

The effects of saturation with the remedy are described as the exanthema iodicum, which shows itself in dark red definite spots of various sizes, spreading over the whole body, remaining without the formation of scales, and disappearing only after a long time; this is called the E. iodicum afebrile, in contradistinction to the E. iodicum febrile, which is seen in syphilitic patients, causes a desquamation of the entire cuticle, and may be considered as a certain criterion of radical cure. The facility with which the iodide of mercury undergoes decomposition has prevented it from being much used internally, as it is apt to produce inflammation of the stomach and bowels, in the same way as iodine itself. In the form of ointment, it is exceedingly useful in promoting absorption; the strength recommended by the author is gr. x to ʒij, (that of the London Pharmacopœia is gr. xv to ʒij,) which quantity is to be daily rubbed into the part affected. The biniodide of mercury is also but little used internally, it is, however, an excellent external application, in the strength of ℈j, to half an ounce of simple ointment, rubbed in three times a day. (The strength of the preparation in the London Pharmacopœia is ʒj to ℥j.)

"The action of this ointment (applied as a plaster spread upon leather) is the following: In the space of half an hour or an hour, varying according to the spot where it is applied, or according to the individual constitution, burning pain comes on which in some persons lasts two or three hours, but in very sensitive subjects even longer. In the third hour the cuticle begins to shrivel, and then rises in little vesicles filled with transparent fluid. They increase in size, and form at length a single bladder, and the fluid becomes opaque. On the second day, the raised epidermis is thicker, the quantity of fluid lessened, and upon the third day the thick and solid epidermis separates in the form of a yellowish white crust. When this has fallen off, the part underneath is seen to be already covered with new epidermis, and entirely healed, but slightly red and sensible. This is the course when the bladder is not opened; when it has been torn, the part becomes painful, and discharges pus for a long time. After three days have elapsed, the application may be made again. The patient may be allowed to walk about during the painful stage, if it be not applied upon the perineum or the lower extremities."

In cases of inflammation of the external tunics of the eye, (blepharophthalmoblenorrhœa,) it may be applied to the eyebrow; the author adds however, "it is of course understood that in this disease, bloodletting, emetics, cold applications, and the sulphate of quinine must not be omitted. I have been fortunate enough by this treatment to save the eye in every case where the patient consulted me sufficiently early." Now in the first place, even when there is ulceration of the cornea, the disease may almost always be cured, if not easily by the above mentioned list of remedies, at any rate by more simple means, and it is no great subject for boasting to be able to cure a complaint on the condition of the patient's applying sufficiently early in the progress of the disorder. In otitis and parotitis it is to be applied behind the ear, acting in a way analogous to the cantharides, but more quickly, and causing rather an effusion of lymph than of serous fluid.

"In bronchocele its powerful effect may be mathematically shown, if the tumour is measured before the application of the plaster; for upon the falling off of the dried cuticle the size is found to have diminished sensibly. In this disease no preparation of iodine is comparable with the binodide of mercury. It performs in one month what the other forms will scarcely do in three or four. I have treated goitres of enormous size with this medicine, and at the first application all the threatenings of suffocation or apoplexy (when they existed) ceased. Soft or so called lymphatic tumours of the thyroid body can be entirely cured, whatever is their size. Those which are indurated, as well as endemic goitres, are harder to cure than those which are soft, and those which occur sporadically." *

Condylomata about the anus and perineum, or even within the rectum and vagina, are cured by this ointment; and unless they are exceedingly extensive and indurated, a single application, although very painful, combined with the internal use of iodine, has been found sufficient. The ioduretted syrup of iron was not found as useful as was expected, for children always refused to take it after a few doses. Iodammonium was administered in cases where nitrogen was thought to be deficient in the urine, in scrofulous subjects; but "every practical physician knows that the signs which are founded upon chemical investigations are extremely uncertain, whilst the human system is something besides a chemical labora-

* In Styria, about 100 miles south-west of Vienna, cases of goitre and cretinism exist to a frightful extent in the population among the hills: bodily deformity and idiotcy with or without bronchocele, are as prevalent, and to as extreme a degree as in Switzerland itself.—Rev.

tory." Lastly, the mineral waters containing preparations of iodine are not unfrequent, existing, as the author remarks, almost in every place where salt is found. Warmth also is, however, required, and the most powerful of all is at Lippich, in Slavonia, containing at a temperature of 140° a grain of iodide of sodium in every pound of water. The cures performed by this water in scrofula, gout, and syphilis are almost incredible.

Instead of giving us the usual history of syphilis and the progress of the disease, our author, in his chapter entitled "The Pathology of Syphilis," lays down what he calls "the articles of his belief," fourteen in number, as the readiest plan of letting us know his ideas. It may be interesting to know what his opinions are upon some of the still debated questions, and therefore we will make a brief abstract of them before we follow him in his practical views.

Dr. M. believes in the justice of the ordinary division into primary and secondary syphilis, and also that the former may remain for a long time a merely local disorder: that of one hundred ulcers in the genital organs, ten at most are true venereal ulcerations: that we possess no characteristic and certain sign of chancre, and that a continued close observation of the course of the complaint can alone decide the question: that the real Hunterian chancre now scarcely exists (owing to an improved mode of treatment,) and that the hard base and thickened edge were formerly almost entirely due to the irritating plan of treatment: that the slow course is an essential particular of the true venereal sore: that there are local syphilitic ulcers possessing an exanthematous and volatile character, which in a few days produce red spots upon the skin, and superficial ulceration of the mucous membrane of the throat, without there being as yet any general lues;—this form readily causes iritis;—if the disease is not at once cured, in the shortest time lues follows, the symptoms of which extend over the whole system: that the inoculation is but an uncertain method of diagnosis, as to its results, and that it increases the danger of constitutional infection: that gonorrhea and syphilis are not different forms of the same disease, although on the other hand they may both be followed by secondary symptoms: that syphilis may be inherited from the father or mother, but more easily from the latter: from a syphilitic mother specific ulceration in various parts, tendency to caries and hydrocephalus are derived; from a mother infected with gonorrhea, glandular swellings, which obstinately resist the usual antiscrofulous remedies: that gonorrhea is entirely distinct from the leucorrhea of chlorotic and scrofulous subjects, because it is always the same, and that when it appears the slightest, there is most frequently metastasis to the testicle or the eye, resembling an exanthematous disease, which, if it is not well developed upon the skin, is liable to attack internal organs; that every case of gonorrhea will get well of itself without any evil consequences; but when suddenly suppressed will produce secondary disease: that one of the abovementioned transient exanthematous chancres cures and entirely disappears after a short time: the patient has, however, incurred the infection of general lues, which becomes developed in his system, and upon his marrying, or indulging in sexual intercourse, excretions are discovered which become true chancres: that this is a matter of great importance, as an innocent person may be accused of being infected with syphilis: lastly, that true chancres may exist within the urethra.

The part of the work on the treatment of these disorders begins with the treatment of gonorrhea, " a disease, like *other* exanthemata, taking its origin from some infection, producing a secretion of matter resembling that from which it originated, and which is of itself curable after this matter has been excreted." There is nothing in this chapter which need detain us, for no new plan of treatment is laid down, nor does he recommend the administration of iodine in any form. The physician should point out to the patient the danger of the secondary diseases which arise if the normal course of this complaint is disturbed. He alludes here to the suppression of the discharge, and allows five or six weeks as the time necessary for the cure. " I consider myself bound in conscience, out of humanity, to warn my colleagues against the use of cubebs and its preparations." He, however, gives the balsam of copaiba after the inflammatory stage has passed.

In the treatment of *strictures of the urethra* he condemns dilatation as prejudicial, and cauterization as perfectly inadmissible. " Strictures and thickening of the coats of the bladder are the same disease, although they produce different symptoms; absorption alone is able to remove these deposits." Besides the ordinary use of bougies for pressure, he applies the ointment of the iodide of mercury to the perineum. In cases of *retention of urine from* stricture he gives two plans, which are always to be had recourse to previous to the performance of the operation of paracentesis vesicæ. The first, which seems to us somewhat hazardous, is the administration of an emetic to produce vomiting; a case, however, is related to illustrate the beneficial action of this remedy. The second plan is a mechanical mode. A smooth and large straight catheter, with an eye at the end, is to be introduced down to the stricture, against which it is to be firmly pressed, and the prepuce is to be held by an assistant against the instrument, so as to prevent the entrance of any air: the surgeon then, by means of a large syringe, which can be accurately adapted to the top of the catheter, injects with a moderate degree of force an ounce of oil, which gradually finds its way through the strictured part and into the bladder. As soon as it reaches the bladder a violent effect to expel the urine is made; when the surgeon draws back the piston of the syringe the oil flows back and is followed by three to five ounces of urine. This is sufficient to relieve the patient for the time, and the operation may be repeated after the lapse of half an hour. He adds, that after the use of this plan for a few days, the urine begins to flow of itself, rendering the application more seldom necessary. The syringe should be capable of containing six ounces.

*Treatment of primary syphilis.* In the treatment of primary sores there is little in this volume requiring remark. His applications to primary sores are only warm bathing and remedies of that nature; with careful attention to the temperature of the body, without any alteration as to diet. In this way he allows three to four weeks for the cure of an ordinary sore. In cachectic or scrofulous subjects, where the powers of the system are scarcely able to perform the excretion of the matter, and the formation of the hard base of the chancre, (which he looks upon as a kind of natural preservative or dam, against the occurrence of secondary symptoms,) some medicine is required to increase the powers without increasing the action of the absorbent vessels, and this is only to be done by the iodide of potassium in large doses.

*Treatment of secondary syphilis by preparations of iodine.* The objects of the treatment are to oppose the destructive agency of the poison upon the function of nutrition, and to excite such activity in the skin as will be sufficient to produce a critical exanthematous eruption; for, he says, at the present day the disease is not as it used to be, an acute exanthema, accompanied with fever, capable of being propagated without sexual intercourse, in the ordinary way of contagious disorders. The only means of performing the cure is by iodine.

*Mercury* often cures both the primary and secondary symptoms, but it occasionally fails. The author exhibits the evils and disadvantages of the mercurial cure under eight heads. The chief of these are—the objections and want of attention of the patient; the difficulty of accurately controlling the action of the medicine; the uncertainty of the period at which its specific effects (salivation) show themselves; the depressing influence of mercury which prevents the patient from continuing sufficiently long with the medicine; the diseases actually brought on by mercury; and its leaving the patient in a weakened and cachectic state.

The advantages of the *iodine cure* are, on the contrary, the following: iodine acts at once on the disease without involving the system; instead of depressing the patient it stimulates his appetite and improves his digestive powers; it produces the wished for eruption of the skin which is critical, indicative of saturation by the remedy and of a perfect cure; salivation occurs from iodine only where mercury has been previously exhibited; the cure is complete in three or four weeks; no relapse takes place after the rash has once appeared; and finally, this remedy may be used in very young, weak, and cachectic individuals.

*Description of a methodical cure by iodine.* A dose of aperient medicine commences the treatment, and the rest consists of the following parts—viz., the regular use of the iodide of potassium and of baths; the local application of a solution of iodine when local affections are present requiring treatment, and a suitable attention to diet and regimen. The iodide is to be administered in doses sufficient to give a powerful impulse to the functions of the nutritive organs, without increasing the activity of the absorbent system, and this can be done by large doses. A patient 15 years of age may begin with a scruple.

℞ Potass. Iodidi. ℈j.

Aquæ destill. vel aq. ceras. nigr. ℥iij.

This quantity to be taken every day before dinner, divided into three doses. The first third early in the morning, and the last about two hours before dinner. After a few days ten grains may be added, and so on up to a drachm, beyond which it is seldom necessary to proceed.

The baths, which appear to be quite as necessary a part of the treatment, are made of iodine, common salt, &c., sufficient quantity of the iodide of potassium to render the iodine soluble. Before Dr. Moj'sisovics commenced the use of the baths, the time occupied in the cure was twice as long. The heat is to be moderate, and they are not to be used in the patient's bedroom, in consequence of the volatilization of some of the iodine rendering the atmosphere unpleasant and irritating to the mucous membrane of the lungs; for the same reason the bath is to be covered with a large cloth, fastened round the patient's neck before the ingredients are added to the water. The strength at first is to be one drachm of iodine, and one and a half of iodide of potassium, with two to three pounds of common salt. The patient should spend an hour in the bath, in which during a part of the time he experiences an almost unbearable burning and itching; he should then return to bed, and remain until the perspiration ceases, when he may get up. In three days the iodine is to be increased to a drachm and a half, and after seven days to two drachms of iodine, and three of iodide of potassium. After the patient is accustomed to the bath some little time, the burning sensation ceases, and this indicates that the solution should be increased. About the tenth or eleventh day a slight febrile action comes on, with thirst and disturbed sleep, and irritation of the skin and a partial scarlet eruption appears. At the fourteenth day it is generally entirely developed, being most clearly seen in the morning. Desquamation begins on the fifteenth and is complete on the seventeenth. This is a sign of a perfect cure. In individuals with thick skins, and of a torpid temperament, the eruption does not appear until the seventeenth day, and the desquamation in the twenty-first. The patient at last may remain an hour and a half in the bath. In one or two corpulent subjects the author observed an eruption resembling herpes zoster, which was exceedingly painful. The hands become discoloured after the fifth and sixth bath, and remain so until the exfoliation of the cuticle.

For a local application a similar solution is used :

℞ Iodin. gr. x.

Iodid. Potass. ℈j.

Aquæ, lb.j. M.

This may be applied to primary and other sores, and may be drawn up the nostrils every two hours in ozæna, and used as a gargle in ulceration of the throat.

The diet requires attention also, for, as Dr. Moj'sisovics observes, in order to try his plan of treatment, it is but just to attend to even the most minute direction. Amylaceous food is not to be taken ; the patient may eat meat and green vegetables.

Iodine is taken into the circulation very quickly. Traces of the iodide of potassium have been found in the urine two hours after the dose of 6⅓ grains had been given, and it was even discovered in the secretion of the ceruminous glands of the external auditory canal. Except for a short time daily during the summer, when there is no wind, the patient should remain in a warm room whilst he is undergoing this treatment.

The last part of the work is composed of twelve cases of primary and secondary syphilitic disease treated on this plan, and according to these the result certainly is most satisfactory. There are also a few cases interspersed in the body of the work, to illustrate particular points of which the author was writing.

If the treatment is as certain and successful as the author declares it to be in his interesting little volume, it will of course be welcome to all practitioners, and its merits will soon be acknowledged. The only practical difficulty is the objection which most patients in this country have to remain so long at home giving up their ordinary occupations, and consider themselves invalids, whilst undergoing treatment for secondary syphilis. In hospital practice there can be no difficulty in putting this mode of treatment in force, and we trust we shall soon hear that this has been done.

